# Supplementary material for: Pancreatic Islet Cells Response to IFNγ Relies on Their Spatial Location within an Islet
Source: Cells. 2022 Dec 28;12(1):113. doi: 10.3390/cells12010113 (PMC9818682; doi:10.3390/cells12010113)
Supplement: Supplementary file 1 [file cells-12-00113-s001.zip › cells-2092783-supplementary.pdf]

Supplementary table S1 : List of primers used for qPCR

Gene	Sequence fw	Sequence rv
Igtp	AGCCCGTCTTTTCACGACTT	TGATGCACTTGTCGCAGACT
Irgm2	CCAACTTCCCTAGCGGTCTC	GAGTGAGTTCCAGGACAGCC
Cxcl10	CCA CGT GTT GAG ATC ATT GCC	TCA CTC CAG TTA AGG AGC CC
Irf1	TCT CGG GCA TCT TTC GCT T	GGG TCT CAT CCG CAT TCG AG
Ifngr1	ACAGCTCTCCGTCCTCGTAT	CACTCCGGTTATGCTCCACA
Ifngr2	TGGGCCAAAGGTGACAAGAA	ATAAGCTTGGATAGCCCGCC
Gcg	TGAAGACAAACGCCACTCAC	TGACGTTTGGCAATGTTGTT
Ins1	CAGAGACCATCAGCAAGCAG	GGGACCACAAAGATGCTGTT
Sst	TCCGTCAGTTTCTGCAGAAGTCTC	GTACTTGGCCAGTTCCTGTTTCCC
Ppia	CAGGTCCTGGCATCTTGTCC	TGCTTGCTGGTCTTGCCATTCC
Spp1	ATC TCA CCA TTC GCA	TGT AGG GAG GAT TGG AGT GAA A
Amy1	GTTATCCGCAAGTGGAATGG	TCGCTGATTGTCATGGTTGT
CD274	GCATTACAAGGCCTACCTGGA	CAGCACCTCAGGGTGACTTC
H2(dlb1q1)	TGCGGACTACAAGCGAATCA	TTCTGGATAACCCTCGGCCT

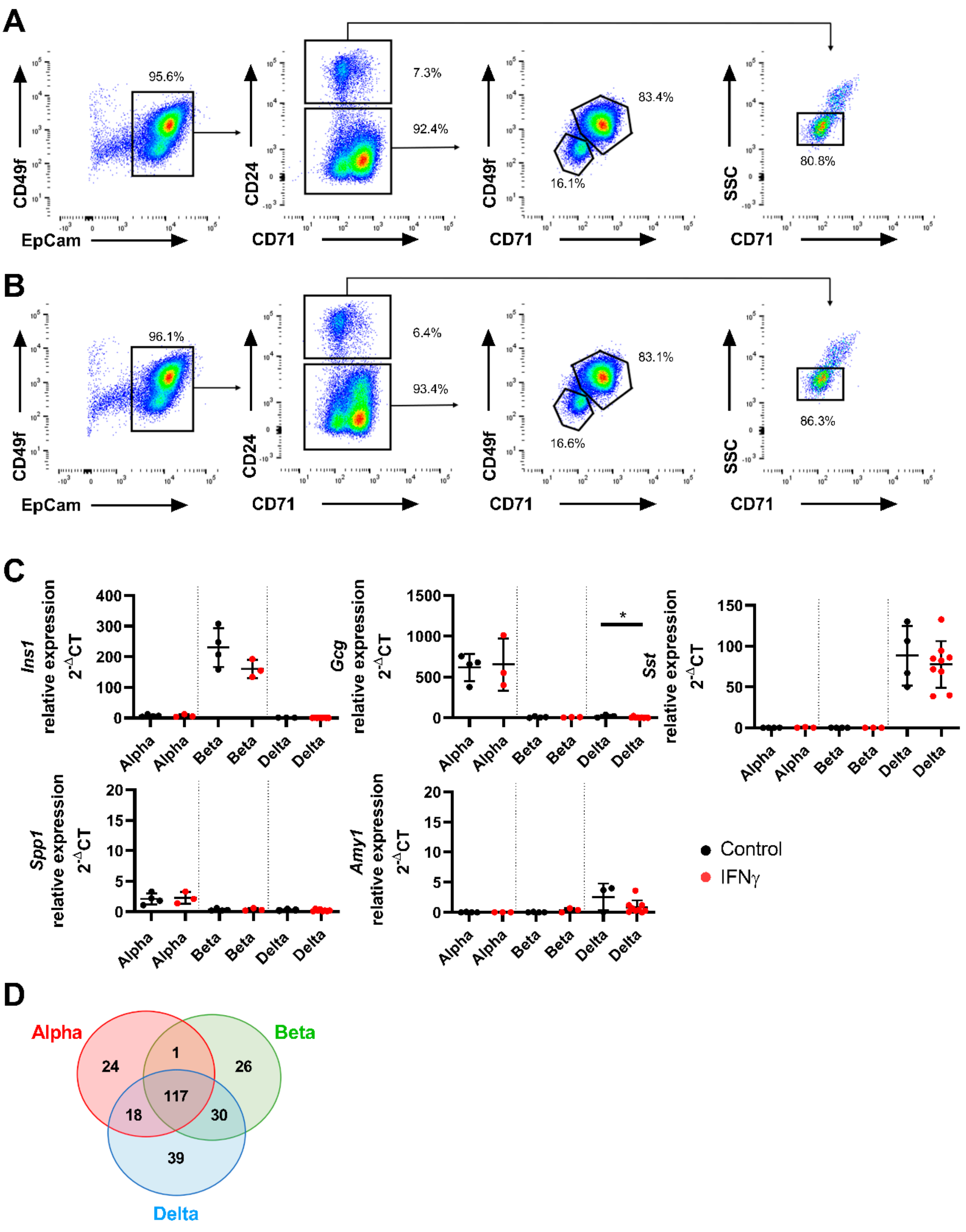

Supplementary Figure S1. Alpha, beta and delta cells sorting strategy and quality. Islets from adult C57BL/6 mice were treated for 20h without (control) or with 5ng.mL^{-1} IFNγ. (A, B) Representative flow cytometry plots of control (A) and IFNγ treated islet cells (B). CD24, CD71 and CD49f expression were analyzed on EpCam+ cells after excluding dead cells (propidium iodide positive cells) and hematopoietic and endothelial cells (lineage [Lin]:CD45-, TER119-, or CD31- positive cells). The EpCam+ fraction was divided into $CD24^{high}$ and $CD24^{low}$ cells (middle left panel). The $CD24^{low}$ fraction was further subdivided into CD71+ and

CD71- cells (middle right panel), respectively enriched for beta and alpha cells, while the CD24^high^ fraction contained delta cells (right panel). (C) Expression of *Ins1*, *Gcg*, *Sst*, *Spp1* and *Amy1* in sorted cells treated (in red) or not (in black) with 5ng.mL^{-1} IFNγ for 20h. n=3-9. (D) Venn diagram presenting the distribution of genes induced by IFNg in alpha, beta and delta cells (ajusted p-value < 0.01). Data are represented as mean ± SD, *p<0.05.

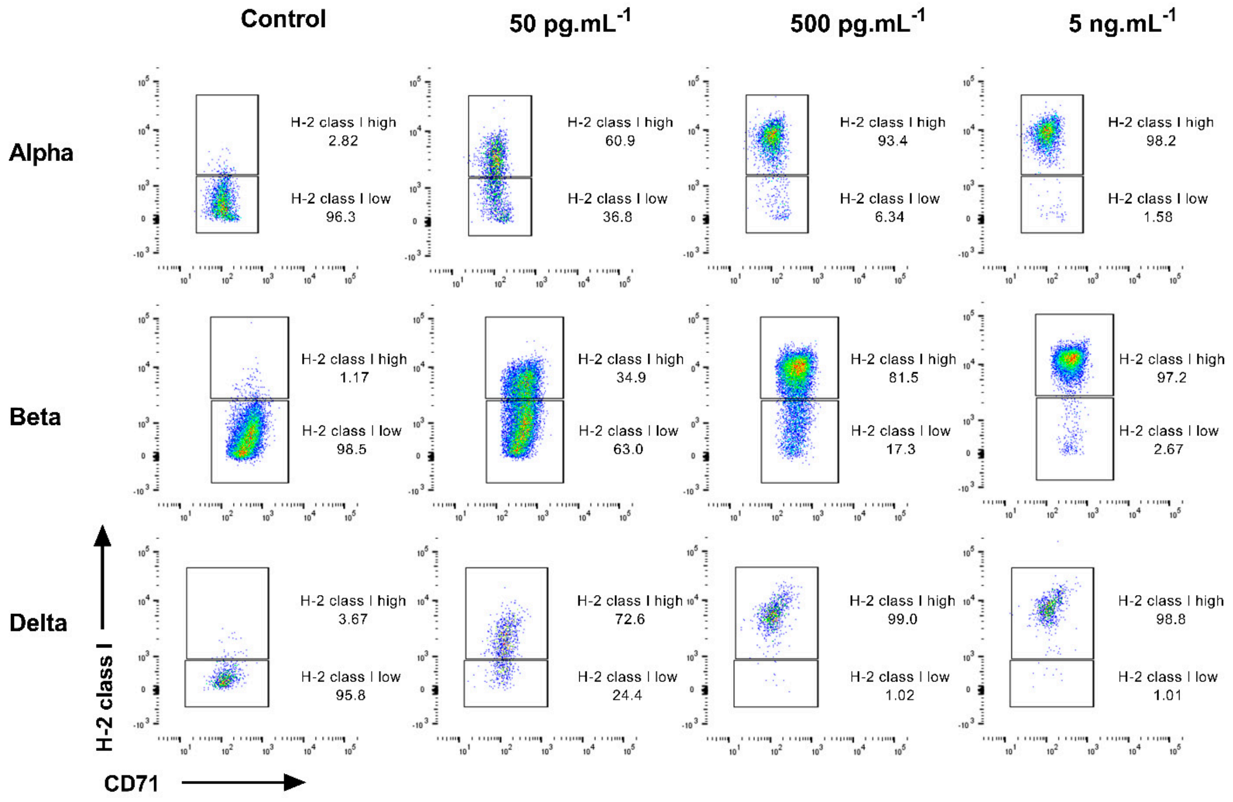

Supplementary Figure S2. Mouse MHC class I expression at cell surface. Representative flow cytometry plots showing H-2 class I surface expression on alpha, beta and delta cells from islets treated with different concentration of IFNγ (0-5ng.mL^{-1}) for 20h.
